# A Short Message Service (SMS) increases postpartum care-seeking behavior and uptake of family planning of mothers in peri-urban public facilities in Kenya

**DOI:** 10.1371/journal.pone.0239213

**Published:** 2020-09-30

**Authors:** Rachel M. Jones, Grace Kimenju, Shalini Subbiah, Amy Styles, Nicholas Pearson, Sathyanath Rajasekharan

**Affiliations:** 1 Department of Research & Design, Jacaranda Health, Nairobi, Kenya; 2 Independent Researcher, Nairobi, Kenya; Baylor College of Medicine, UNITED STATES

## Abstract

**Background:**

It is estimated that one third of maternal deaths in Kenya in 2014 could have been prevented by more timely care-seeking. Mobile health interventions are increasingly being recognized as tools for the delivery of health education and promotion. Many maternal deaths occur in the first few weeks after delivery and mothers who are given adequate care in the postpartum period have better health outcomes. Kiambu County, Kenya has a high level of literacy and phone ownership amongst mothers delivering in public hospitals and was chosen as a site for a postpartum short message service intervention.

**Methods:**

Women were recruited after delivery and randomized to receive a package of mobile messages or standard of care only. Messages covered danger signs, general postpartum topics, and family planning. Endline phone surveys were conducted at 8 weeks postpartum to assess knowledge, care seeking behavior and family planning uptake. Analysis was conducted using Stata and is presented in odds ratios.

**Results:**

Women who received the danger sign messages were 1.6 times more likely to be able to list at least 1 danger sign and 3.51 times more likely to seek treatment if they experienced postpartum danger signs. There was no significant difference in routine postpartum care seeking or care seeking behaviors concerning newborns. Women who received family planning messages were 1.85 times more likely to uptake family planning services compared to controls and 2.1 times more likely to choose a long-acting method.

**Conclusions:**

Simple, low-cost mobile interventions can support women in the early postpartum period when the information is targeted to particular points in the postpartum continuum. Additional research is needed to understand the interplay between healthcare providers and mobile health interventions. Health policy makers should consider direct mobile interventions for women as an option for supporting positive maternal health outcomes in certain populations.

## Introduction

Despite the global progress over the last two decades, future strategies for reducing maternal and neonatal mortality will require an expansion of the “solution space” that support quality health systems [1]. Mobile health (mHealth) interventions are increasingly being recognized as tools for the delivery of health education and eliciting changes in care-seeking behaviors. In Kenya, nearly 89% of the population overall—and 63% of the population in the lowest wealth quintile—have access to mobile phones, and recent literature has shown that mHealth interventions are feasible and acceptable for transmitting information about pregnancy and birth [2, 3]. However, there are limited studies that evaluate the impact of mHealth interventions on maternal health care-seeking [4–6].

Maternal mortality in Kenya is 362 maternal deaths per 100,000 live births, a rate 57 times higher than in Western Europe [7, 8]. The 2014 Confidential Enquiry into Maternal Deaths (CEMD) by the Kenya Ministry of Health found that 33% of the maternal deaths reviewed could have been prevented by more timely care-seeking [9]. Many of these deaths occur in the first few weeks after delivery, and mothers who are given adequate care in the postpartum period would have better health outcomes [8, 10, 11].

There are significant gaps in quality of care for mothers in Kenya during the postpartum period. In the critical 6 weeks after delivery, 43% percent of women in Kenya do not get a postnatal checkup for their health [7]. A lack of awareness of and knowledge about postnatal care (PNC), and cultural factors after birth have been cited as reasons for low postnatal checkup attendance for mothers’ health [12–14]. Knowledge of postpartum danger signs is tied to women’s decision to seek care [15]: a recent study conducted in Kericho County, Kenya found that only 10% of pregnant women could name two or more danger signs that might occur after delivery [16].

Inadequate care during the postpartum period also results in a high unmet need for postpartum family planning (PPFP). Only 51% percent of women in Kenya access a modern method of family planning during the postpartum period [17], and short birth spacing is common with 50% of non-first births in Kenya born within 24 months of a previous birth [18]. These factors put mothers at higher risk for morbidity and newborns at higher risk for morbidity and mortality [19]. Neonatal mortality is estimated at 21 deaths per every 1,000 live births, a rate seven times higher than in Western Europe [20].

Here we present the results from a randomized controlled trial (RCT) that evaluated the impact of short message service (SMS) messaging on postpartum care-seeking behaviors of mothers in resource-limited settings in Kenya (client category 1.1 under the World Health Organization (WHO) classification of digital health interventions [21]). We investigated whether a discrete series of SMS messages sent to mothers during the first 6 weeks postpartum would result improve knowledge, identification of complications and care-seeking behaviors, including family planning uptake. Our key results demonstrated that the messages are associated with significant improvements in maternal knowledge of maternal danger signs, care-seeking after health potential danger signs appeared, and uptake of modern family planning methods by 8 weeks postpartum. However, the SMS did not increase general postpartum or postnatal care seeking behaviors. To our knowledge, this is the first experiment that seeks to evaluate the impact of an SMS intervention on several outcomes in the early postpartum period by comparing different messaging packages.

## Methods

### Study setting

Study participants were recruited from postnatal wards in three public health facilities in Kiambu County, Kenya, which is located 13–31 kilometers north of Nairobi City County. Facilities were selected on the basis of providing maternal and infant health services to the surrounding neighborhoods; were considered ‘mid-volume’ as they averaged at least 300 deliveries per month based on the Kenya Health Information System; and served populations of both semi-rural and peri-urban centres. The final facilities selected for the study were Kihara Sub-District hospital, Ruiru Sub-District hospital and Tigoni District hospital.

### Study population

Women between 18 and 40 years old were eligible for inclusion if they had delivered vaginally at one of the three study sites and had access to a mobile phone with which they could receive SMS messages. Women were not eligible for inclusion if they had complications in their delivery (including undergoing a cesarean section) or if their infants showed signs of illness/complications prior to discharge, such as jaundice, abnormal breathing, or heart conditions. The list of complications that excluded women from participation was compiled by an obstetrician and defined as complications that could require specialized care. Research assistants approached all eligible women being discharged (~24 hours after delivery) from the facility and gave them information describing the study. Potential participants were taken through a voluntary, written informed consent process and signed an informed consent form if they chose to enroll in the study. Women could withdraw from the study at any time and stop the messages by sending the word “STOP” to the shortcode from which they received messages.

### Study design

**Fig 1:**
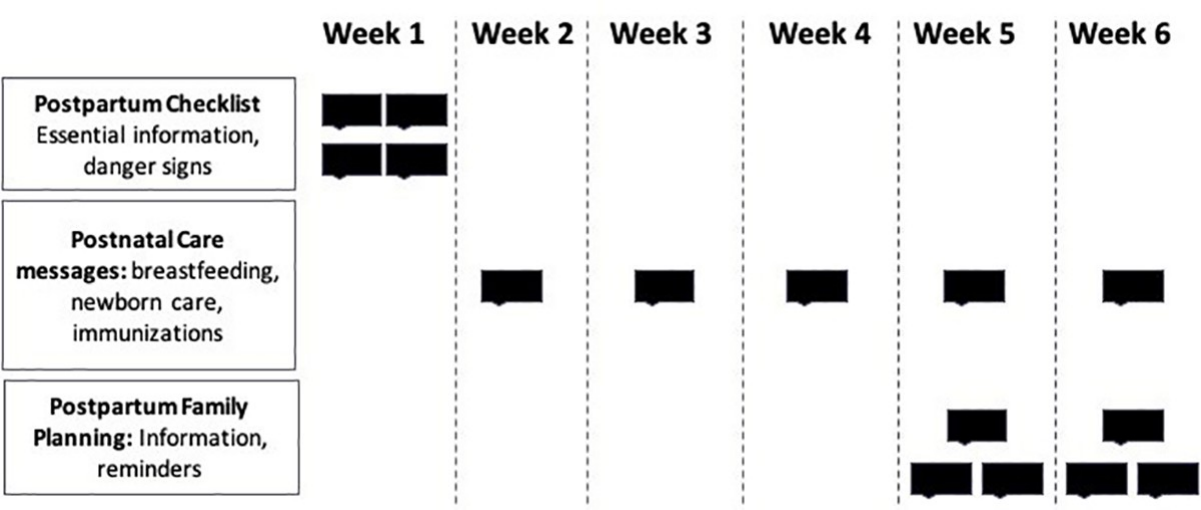
Message packages and timing.

A randomized controlled trial was conducted with four study arms. Following enrollment, participants were randomized at the individual level into one of four arms and uploaded into the SMS system by a research assistant not involved in recruitment or enrollment. Randomization was done by utilizing a random number generator in Stata (2009 StataCorp, College Station, TX) to create a list of numbers 1 to 1000 in a random order. The numbers 1–250 were assigned to the 1^st^ study arm, 251–500 assigned to the 2^nd^ study arm, 501–750 assigned to the 3^rd^ arm, and 751–1000 assigned to the 4^th^ Arm. The random number list (and associated study arm number) was copied into the enrollment sheet. As baseline surveys were completed, the phone numbers were copied in the exact order of completion into the enrollment sheet and assigned the corresponding study arm based on the random number in the column next to it. Research assistants involved in recruitment and enrollment were not aware of the number allocation. The phone numbers were then uploaded into the SMS system by group. Arm 1 was the control group (Control), where participants were enrolled in the study but only received the current standard of care (no SMS). Each of the three intervention arms had a different combination of SMS content. Arm 2 received the “postpartum checklist” (PPC) the week following discharge, which consisted of Yes/No questions regarding their postpartum state. Arm 3 received the “postpartum checklist” plus general postnatal care messages and reminders in the 4 weeks after discharge (PPC+PNC). Arm 4 received the “postpartum checklist” plus family planning messages/reminders between 4 and 6 weeks after discharge (PPC+FP). At the time of enrolment, all women were informed about the randomization process, and that there was a possibility of placement in the control group. A visual of the message timing for the different intervention arms can be viewed in **Fig 1**.

### Message packages, design, and adaptation

All SMS were sent in Swahili and were completely free for the participants to receive and to respond to, with the intention of lowering accessibility barriers for low-income women. All messages were developed using international guidelines and academic publications with input from clinicians at Jacaranda Health.

#### Postpartum Checklist (PPC) messages

The components of the checklist and their sources are described in McConnell et al, 2016 [22]. New mothers received four messages per day, on days 2, 3 and 4 post-discharge. Each message was in the form of a yes/no question and was designed to prompt mothers to self-assess for maternal infection, secondary postnatal hemorrhage, postnatal preeclampsia, insufficient breastfeeding, jaundice in newborns, and local infection. At the end of each set of messages the mother is encouraged to indicate if she has observed the described symptoms in herself or in her baby. If the mother indicated that she observed the symptoms described in the messages, she received a referral message encouraging her to visit the hospital.

#### General Postnatal Care (PNC) messages

The PNC message stream included 11 health messages and were sent every 3 days, starting at day 6 up to day 36 after discharge. The messages included information related to breastfeeding, baby care, and family planning. The messages also encouraged women to adhere to routine postnatal follow-up care and the baby’s immunization schedule.

#### Family Planning (FP) messages

The FP message stream included 6 family planning messages, began 30 days after discharge, and continued every three days through day 45 after discharge. The messages included information related to healthy birth spacing of 2 years, modern methods of contraception, and a reminder that one can get pregnant again soon after birth before having periods.

#### Message design and adaptation

All message packages were tested through a series of focus groups in Kiambu County, each with 8–12 postpartum women who delivered at public hospitals. The focus groups first probed for the types of information women felt they needed after delivery and combined this with information that was prioritized by clinicians. The subsequent focus groups then tested the messages for language clarity and understanding, preferences around timing/frequency of messages, and preferences around language choice.

#### Sample size

We aimed to recruit 900 women in order to have 80% power in detecting a 10% change in postnatal care attendance among mothers, while also accounting for an 18% loss to follow up.

### Data collection

Enrollment took place over 5 months, beginning in November 2017 and concluded in March 2018. Research assistants approached all eligible women on the day they would be discharged from the facility and gave them information describing the study. If a woman consented to participate, she completed the in-person baseline survey administered by a research assistant at the hospital before discharge and was enrolled in the study. Endline survey data was collected 8 weeks after discharge through a phone survey. Endline data collection concluded in May 2018. All hospitals were enrolling simultaneously during the enrollment period. Survey data were recorded on a tablet using SurveyCTO software (2016 Dobility, Inc, Cambridge, MA). Calls were attempted 4 times before the participant was deemed unreachable.

### Data analysis

Data was analyzed in STATA 11 (2009 StataCorp, College Station, TX). Outcomes related to danger sign knowledge and care seeking related to danger signs compared all participants who received the postpartum checklist (PPC group, PPC+PNC group, PPC+FP group) together versus the control group who did not receive any messages. Outcomes related to general postnatal care (immunization, mother’s postpartum checkup, and child wellness visits) compared women who received the PPC+PNC messages versus all other participants grouped together (PPC, PPC+FP, Control). Outcomes related to family planning (uptake of family planning, planning to uptake family planning, LARC method use) compared women who received the PPC+FP messages versus all other participants grouped together (PPC, PPC+PNC, and Control).

#### Statistical analyses

All demographic data was described using Pearson’s chi2 tests. Univariate logistic regression models were run, followed by mulitvariate logistic regression models controlling for potential confounders. Outcomes are reported as Odds Ratios (OR) with their 95% confidence intervals (95% CI).

### Ethics statement

Ethical clearance for the study was obtained by the Amref Ethics and Scientific Review Committee in Kenya under protocol number P355-2017. Interested participants provided written informed consent and were alerted to both the potential benefits and risks, as well as the fact that they may be randomized to not receive messages. At all points during the study, participants could voluntarily decide to withdraw from the study or opt out from receiving the SMS messages by sending the word ‘STOP’.

## Results

**Fig 2:**
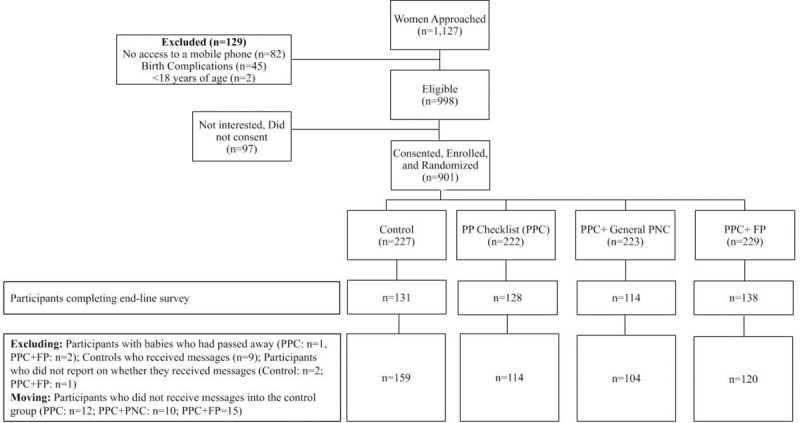
Flow chart of participant enrolment and study arm allocations.

Study participants were equally allocated to each of the four arms of the study, resulting in 225 women in each arm. In total 1,127 women were approached for potential inclusion in the study. At the end of recruitment there were 901 new mothers enrolled in the study, and we were able to collect endline data from 511 women (57% of those who completed baseline). Reasons for ineligibility included: participants not having access to a mobile phone (n = 82), complications relating to birth such as prematurity, stillbirth, infant death, home delivery or cesarean delivery (n = 45), and age (n = 2, <18 years of age). Of the potential participants who were eligible but did not consent, the majority indicated they were not interested in the study (n = 86) with no other reason before the consent process, 6 participants went through the consent form but refused their consent, and 5 women they wanted to consult with their partner/husband before consenting. Loss to follow-up occurred when participants were not reached or were not able to complete the endline survey after four separate attempts via phone (**Fig 2**).

During the endline survey participants were asked if they remember receiving SMS messages. We excluded: 9 participants who indicated that they had received SMS messages despite being in the control group; and 3 participants who did not report if they had received messages or not. In addition, some participants in the PPC (12 individuals), PPC+PNC (10) and PPC+FP (15) arms indicated that they had not received SMS messages. We moved these 39 individuals to the control group. Two additional participants were excluded since their baby was deceased at the time of the endline survey (**Fig 2**).

### Demographics

There was no significant difference between demographic characteristics of participants in the control group compared to clients who received the PPC messages (age, education, marital status, shared toilet as an indicator of socio-economic position [23], parity). **Table 1** presents characteristics of the participants, disaggregated by group.

**Table 1:** Demographic characteristics by group.

	Control (N = 159)	PPC (N = 114)	PPC+PNC (N = 104)	PPC+FP (N = 120)	Significant (S) / Not Significant (N.S.)
Age—mean (SD)	25.7 (5.0)	26.2 (5.2)	25.6 (5.4)	25.4 (5.2)	N.S.
Education—N (%)					N.S.
Primary	39 (24.5)	34 (29.8)	26 (25.0)	22 (18.3)	
Secondary	94 (59.12)	58 (50.9)	54 (51.9)	64 (53.3)	
Higher	26 (16.35)	22 (19.3)	24 (23.1)	34 (28.3)	
Married—N (%)	133 (83.6)	94 (82.5)	85 (81.7)	100 (83.3)	N.S.
Shared Toilet—N (%)	104 (65.4)	77 (67.5)	72 (69.2)	70 (58.3)	N.S.
Parity—N (%)					N.S.
First Pregnancy	53 (33.33)	42 (36.8)	46 (44.2)	46 (38.3)	
Second Pregnancy	63 (39.62)	49 (43.0)	32 (30.8)	43 (35.8)	
Third+ Pregnancy	43 (27.04)	23 (20.2)	26 (25.0)	31 (25.8)	
Received Any ANC—N (%)	154 (96.9)	112 (98.2)	104 (100)	118 (98.3)	N.S.
Timing of First ANC Visit					
First Trimester	19 (11.95)	8 (7.0)	6 (5.8)	11 (9.2)	N.S.
Second Trimester	82 (51.57)	46 (40.4)	54 (51.9)	67 (55.8)	
Third Trimester	58 (36.48)	60 (52.6)	44 (42.3)	42 (35)	
Four or more ANC visits—N (%)	106 (68.8)	71 (63.4)	71 (68.3)	86 (72.9)	N.S.
Hospital Attended—N (%)					N.S.
Kihara	58 (36.5)	36 (31.6)	40 (38.5)	49 (40.8)	
Ruiru	39 (24.5)	35 (30.7)	28 (26.9)	31 (25.8)	
Tigoni	62 (39.0)	43 (37.7)	36 (34.6)	40 (33.3)	

### General care-seeking behavior for newborn and maternal health

We analyzed the general care-seeking behavior of mothers, defined as situations where women sought routine care but there were no reported complications or perceived danger signs for themselves or their infants. Outcomes related to PNC (immunization, child wellness visits, mother’s postpartum checkup) compared women who received the PPC+PNC messages (n = 104) to all other participants who did not receive PNC messages grouped together (Control, PPC, PPC+PF, n = 393). At 8 weeks postpartum, 100% of participants reported their child had been vaccinated, 99.0% of all participants were exclusively breastfeeding their baby, and 99.8% had brought their child back for at least 1 child wellness visit. There was no significant difference between the women who received PPC+PNC messages and controls in vaccination, exclusive breastfeeding, or child wellness visits. In addition, 98.8% of all participants reported that they had been told by their healthcare provider to bring their baby in for child wellness visits.

There was no statistically significant difference in general maternal care-seeking behavior between the women who received PPC+PNC messages and controls. At 8 weeks postpartum, only 50.0% of women who received PPC+PNC messages reported returning to a facility for a maternal postpartum checkup compared to 52.4% of controls (p = ns). However, if a woman was told by her healthcare provider to come back for a postpartum checkup (51.0% of PPC+PNC message group and 57.25% of controls), she was 14 times more likely to come back to the facility (OR = 14.38, 95%CI: 9.07–22.08).

### Knowledge of newborn and maternal danger signs

Outcomes related to the knowledge of danger signs compared all participants in the intervention arms who received the PPC (PPC, PPC+FP, PPC+PNC, n = 338) grouped together versus the control group (n = 159) who did not receive any PPC messages. The majority of participants (82.9%) were able to list at least 1 danger sign for newborns and there was no statistically significant difference between the control group and women who received the PPC. 35% of all participants’ newborns experienced negative health symptoms in the first 2 months of life, and 85% of those who experienced symptoms sought advice or treatment for their newborn. There was no significant difference between the control and PPC groups. **Table 2** presents the univariate and multivariate odds ratios for these results.

**Table 2:** Newborn postpartum danger sign knowledge & care-seeking behavior.

	Control (N = 159) N (%)	PPC (PPC, PPC+FP, PPC+PNC) (N = 338) N (%)	Univariate OR OR (95% CI)	Multivariate OR*OR (95% CI)
At least 1 newborn postpartum danger sign listed	128(80.5)	284(84.02)	1.27 (0.78–2.07)	1.24 (0.73–2.07)
Newborn experiencing illness in the last 2 months	60 (37.7)	116 (34.3)	0.86 (0.58–1.27)	0.83 (0.55–1.24)
Sought advice/treatment for postpartum danger signs symptoms	56 (83.58)	107 (86.29)	1.23 (0.54–2.81)	1.41 (0.59–3.36)

*controlling for whether a healthcare worker told the client to take up FP services, having used FP in the past, age, marital status, education, SES, number of previous pregnancies, number of ANC visits made, hospital.

After controlling for potential confounders, women who received the PPC messages were 1.6 times more likely to be able to list at least 1 postpartum danger sign (OR = 1.60, 95% CI: 1.07–2.38) compared to control group. Specifically, they were 2.57 times more likely to list fever/chills, a danger sign for postpartum infection or sepsis (95% CI: 1.10–5.96). There was no statistically significant difference between the control and groups that received the PPC messages with respect to the number of women who experienced postpartum danger signs by 8 weeks; however, if a women had received the PPC messages and experienced a postpartum danger sign, they were 3.51 times more likely to seek treatment (95%CI: 1.22–10.07) compared to control. These results are shown in **Table 3** below.

**Table 3:** Maternal postpartum danger sign knowledge & care-seeking behavior.

	Control (N = 159) N (%)	PPC (PPC, PPC+FP, PPC+PNC) (N = 338) N (%)	Univariate OR OR (95% CI)	Multivariate OR* OR (95% CI)
Knowledge of at least 1 maternal postpartum danger sign	83 (52.20)	214 (63.31)	1.58 (1.07–2.31)	1.60 (1.07–2.38)
Fever/chills named as a known danger sign	7 (4.4)	36 (10.65)	2.59 (1.12–5.95)	2.57 (1.10–5.96)
Experienced postpartum danger signs symptoms	25(15.72)	43 (12.72)	0.78 (0.46–1.33)	0.91 (0.52–1.59)
Sought advice/treatment for postpartum danger signs symptoms	9 (28.12)	30 (51.72)	2.73 (1.08–6.91)	3.51 (1.22–10.07)

*controlling for whether a healthcare worker told the client to take up FP services, having used FP in the past, age, marital status, education, SES, number of previous pregnancies, number of ANC visits made, hospital.

### Family planning outcomes

Outcomes related to FP (uptake of family planning, planning to uptake family planning, LARC method use) compared women who received the PPC+FP (n = 120) versus all other participants grouped together (PPC, PPC+PNC, Control, n = 377). At 8 weeks postpartum, 47.9% of all women were on a FP method (57.5% among women who received FP messages, and 44.8% of controls). After controlling for whether their health care provider had told them about FP, and whether they had previously used FP, along with other demographics, women who received FP messages were 1.85 times more likely to uptake family planning services compared to controls (OR 1.85, 95% CI 1.16–2.94). Women who were told by their healthcare provider about FP were 2.27 times more likely to uptake family planning, compared to those who were not told (OR 2.27, 95%CI 1.53–3.35).

Women currently using a FP method were asked what type of FP they were currently using. Women who received FP messages were 2.1 times more likely to use a Long Acting Reversible Contraceptive (LARC) method (implant or intrauterine device (IUD))—compared to controls (OR 2.10, 95% CI 1.06–4.15). The FP results are displayed in **Table 4** below.

**Table 4:** Family planning behavior.

Family Planning Use	Control (Control, PPC, PPC+PNC) (N = 377) N (%)	PPC + FP group (N = 120) N (%)	Univariate OR OR (95% CI)	Multivariate OR* OR (95% CI)
Currently using FP	169 (44.8)	69 (57.5)	1.66 (1.09–2.52)	1.85 (1.16–2.94)
Planning to take up FP	138 (77.1)	37 (86.1)	1.83 (0.72–4.64)	2.04 (0.69–6.02)
LARC Methods	24 (6.4)	15 (12.5)	2.10 (1.06–4.15)	2.05 (1.01–4.16)

*controlling for whether a healthcare worker told the client to take up FP services, having used FP in the past, age, marital status, education, SES, number of previous pregnancies, number of ANC visits made, hospital.

## Discussion

Our results show that a discrete series of SMS messages sent to mothers who delivered at public hospitals is associated with increased postpartum and postnatal health knowledge and an increase in specific postpartum care-seeking behaviors. The ability of a mother to self-identify potential danger signs is important when these signs manifest post-discharge, away from the supervision and immediate advice of health professionals. Mothers who received SMS messages and experienced postpartum danger signs were 3.51 times more likely to seek care for those danger signs than those who did not receive the SMS messages. The early postpartum period can also result in unplanned pregnancies. When women adequately space pregnancies with modern family planning methods, the outcomes are more positive for both the mother and her children [19]. Mothers who received the SMS package with nudges and information on family planning were nearly twice as likely to take up a modern family planning method by 8 weeks postpartum and more than twice as likely to choose a long-acting method, compared to mothers in the control group. Mothers in the intervention groups were also significantly more likely to name danger signs such as fever/chills. As a potential sign of infection or sepsis, which accounts for 15% of maternal deaths in Kenya annually [24], fever/chills in women after delivery are a danger sign that warrants care. It is possible that many women previously assumed that if one experienced fever/chills after delivery that they were unrelated to childbirth. Taken together, these results indicate that a package of SMS messages can increase health knowledge around the postpartum period and can contribute to care-seeking behavior that could save a mother’s life.

In contrast, we were unable to detect a difference between treatment and control groups with respect to postnatal care: knowledge of newborn danger signs, care seeking for newborns with danger signs, and routine child wellness visits. It is important to note that the levels of knowledge and care-seeking were high in the control group (>80%), which may indicate a baseline level of awareness of postnatal care in this population. Health providers may focus more on newborn danger signs than on maternal danger signs when providing information, or new mothers may be especially focused on observing the health of their newborns. We also did not observe differences between groups in increasing the care-seeking for a postpartum (maternal) checkup in the 8 weeks after delivery.

Upon reflection, the messaging around postpartum checkups was broad, did not specify the timing of when a mother should return, or explain *why* postpartum care was imperative (beyond that it was “important” for her health). In contrast, Oramisi and colleagues were able to observe a decrease in failure-to-attend rates for postpartum visits at two weeks with targeted appointment reminders via SMS at a similar Kenyan hospital [25]. The team has since modified the PNC messages by providing actionable steps expected of the user at a specific time. For example, a general PNC message sent to the PPC+PNC group was “To ensure the health of you and your baby, it’s very important that you visit the PNC clinic and baby clinic as soon as possible”. This has been adapted to: “It is very important that you return to the hospital two weeks after delivery or on your appointment date for postnatal care. A healthy mom is a gift to her family! Ask all your questions to your health provider.” The family planning messages, on the other hand, did influence the odds of uptake by 8 weeks postpartum, and the postpartum checklist supported knowledge and care-seeking. Future programs may consider a package of messages that includes the postpartum checklist to identify danger signs, more specific postnatal messages, and family planning messages in order to support women across the postpartum period.

An interesting finding was the strong link between a mother’s positive care-seeking behavior and her recollection of receiving this advice from a health provider. This was the case for uptake of postpartum family planning, postpartum visits for the mother’s health, and child welfare visits. We believe these results demonstrate the use-case for mobile health tools such as those in the study as being a complementary solution that augments provider interactions, and not as a tool to replace the interaction.

Other studies have explored the impact of SMS interventions on early postpartum behaviors. Unger et al (2018) reported increased rates of exclusive breastfeeding and postpartum contraceptive use at 16 weeks postpartum amongst women at a facility in Nairobi, Kenya [26]. Their SMS intervention started in pregnancy and tested both one and two-way messaging. Our study complements their work by suggesting it may be possible to begin an SMS intervention after delivery and still change family planning uptake by 8 weeks postpartum. Additionally, the work by Harrington et al (2019) explored postpartum family planning uptake amongst women in Kisumu county, Kenya using a two-SMS intervention that involved communication with a nurse [27]. Our study indicates that a less resource-intensive mobile health intervention may result in similar positive care-seeking behaviors. Many digital tools are also targeted at lay cadres operating in the community who use the information to improve maternal knowledge and care-seeking and to increase referrals for further care [28, 29]. Our study suggests that it is possibly to directly engage women and influence care-seeking using mobile health tools.

Following the initial analysis of these results, steps were taken to increase access to the messaging platform for women in Kenya. By May 2020, over 150,000 pregnant women and new mothers had enrolled to receive messages from the service (now called ‘PROMPTS’), which is operating in 5 counties in Kenya, including Kiambu County where the study took place. Given the direct method of engaging mothers, this solution is only appropriate for women who have access to mobile phones. Ownership and access to phones in Kenya is increasing [3]. We conduct routine follow-up surveys of the enrolled population (data not shown), and confirm that mobile phone ownership is high amongst the enrolled population, with a small percentage of the population using a partner or neighbor’s phone to enroll in the service. However, the majority of users (>50%) have feature phones, which do not allow for internet access. In addition, women with smartphones had unreliable data access, and prefer information via text message rather than using an internet messaging (IM) technology such as WhatsApp. We believe that in the near term, SMS, rather than IM tools, will remain the most effective means of reaching populations in resource limited settings.

Our study had a few limitations. Given the loss to follow up, the study was not sufficiently powered to find a difference between each of the intervention groups for the outcomes of interest. We were able to strengthen the effect by re-grouping the comparison groups based on who received messages related to the outcomes, versus those who did not receive the messages related to the outcomes. For example, we compared participants who received the PPC messages with the control group for outcomes related to danger signs; we compared participants who received general PNC messages with all participants who did not receive PNC messages; and we compared participants who received FP messages with all participants who did not receive FP messages. We reasoned that this re-structuring of comparison groups based on outcomes was appropriate given that there was no dilution of messages from one intervention group to another. For example, participants not in the PPC+PNC groups did not receive any general PNC care seeking information. In addition, participants self-reported their care seeking behavior and health outcomes at 8 weeks postpartum, which could have resulted in a level of desirability bias during phone surveys. The study was also limited to a short period after delivery so we were not able to analyze FP continuation/discontinuation, exclusive breastfeeding for 6 months, or the full suite of immunization coverage for the children. The loss to follow up was high in our sample as well, which may have influenced the final results. Other phone surveys conducted by Jacaranda Health with similar populations of pregnant or postpartum women in Kenya show a similar response rate (45–50%). Possible reasons for loss to follow up include participants traveling out of network, participants sharing phones with family members and thus being away from their phone at the time of the survey, participants not answering calls from unknown numbers, participants losing or changing phone lines after enrollment, and, in a few cases, participants not having the time or interest/motivation to complete the survey. To decrease loss to follow up, future SMS studies may consider requesting alternative phone numbers at the time of enrollment which can be used if the primary number becomes unreachable. Future studies could also consider making the time to follow up shorter, and having participants save the study phone number in their phones so they are able to recognize the caller at the time of follow up.

Finally, our findings represent a population of women who attended public hospitals for delivery in one county outside of Nairobi, Kenya. Although phone access amongst potential participants was high at 93%, the 7% of women who were ineligible because they did not have phone access may have also been disadvantaged in other ways. These women would be better supported through alternative approaches to information-sharing and reminders, such as via community health workers. Only a small number of potential participants (fewer than 5) in this sample were illiterate or had delivered outside the facility. While several peri-urban areas in Kenya contain similar characteristics to our population in their levels of phone ownership, literacy, and facility delivery rates, generalizability remains limited. Other counties in Kenya have unique language and cultural contexts which could affect the impact of the messages on knowledge and behavior. Additionally, the quality of care received at the health facilities and level of involvement/instruction from health providers could change the perception of the intervention in different settings. We expect the results could therefore vary depending on such factors.

## Conclusions

A simple, low-cost SMS interventions targeted to particular time points in the postpartum continuum can support women to increase knowledge around danger signs, to seek care for danger signs, and to initiate postpartum family planning. Additional research is needed to understand the interplay between healthcare providers and mobile health interventions and to explore ways of increasing routine maternal health checks after delivery, including by increasing communication during the antenatal period. Health policy makers should consider direct SMS interventions, adapted to the local context, for women as an option for supporting positive maternal health outcomes in populations where there is high mobile phone penetration.
